# PTEN: Tumor Suppressor and Metabolic Regulator

**DOI:** 10.3389/fendo.2018.00338

**Published:** 2018-07-09

**Authors:** Chien-Yu Chen, Jingyu Chen, Lina He, Bangyan L. Stiles

**Affiliations:** ^1^Department of Pharmacology and Pharmaceutical Sciences, School of Pharmacy, University of Southern California, Los Angeles, CA, United States; ^2^Department of Pathology, Keck School of Medicine, University of Southern California, Los Angeles, CA, United States

**Keywords:** PTEN, PI3K, AKT, cancer, metabolism

## Abstract

Phosphatase and Tensin Homolog deleted on Chromosome 10 (PTEN) is a dual phosphatase with both protein and lipid phosphatase activities. PTEN was first discovered as a tumor suppressor with growth and survival regulatory functions. In recent years, the function of PTEN as a metabolic regulator has attracted significant attention. As the lipid phosphatase that dephosphorylates phosphatidylinositol-3, 4, 5-phosphate (PIP_3_), PTEN reduces the level of PIP_3_, a critical 2nd messenger mediating the signal of not only growth factors but also insulin. In this review, we introduced the discovery of PTEN, the PTEN-regulated canonical and nuclear signals, and PTEN regulation. We then focused on the role of PTEN and PTEN-regulated signals in metabolic regulation. This included the role of PTEN in glycolysis, gluconeogenesis, glycogen synthesis, lipid metabolism as well as mitochondrial metabolism. We also included how PTEN and PTEN regulated metabolic functions may act paradoxically toward insulin sensitivity and tumor metabolism and growth. Further understanding of how PTEN regulates metabolism and how such regulations lead to different biological outcomes is necessary for interventions targeting at the PTEN-regulated signals in either cancer or diabetes treatment.

## Key concepts

- Phosphatase and Tensin Homolog deleted on Chromosome 10 (PTEN) is a dual phosphatase with both protein and lipid phosphatase activities.
- PTEN reduces the level of PI-3, 4, 5-P_3_, a critical 2nd messenger mediating the signal of not only growth factors but also that of insulin.
- In addition to the canonical PI3K/AKT signaling, PTEN also functions in the nucleus.
- PTEN regulates signals in metabolic regulation, includes the role of PTEN in glycolysis, gluconeogenesis, glycogen synthesis, lipid metabolism as well as mitochondrial metabolism.
- PTEN and PTEN regulated metabolic functions act paradoxically toward insulin sensitivity and tumor metabolism and growth.

## Introduction

PTEN (phosphatase and tensin homolog deleted on chromosome 10) (also named MMAC1/TEP1) was discovered in 1997 independently by three laboratories as a tumor suppressor of which the expression is often lost in tumors (1–3). Later studies established that PTEN is a negative regulator of a major cell growth and survival signaling pathway, namely the phosphatidylinositol-3-kinase (PI3K)/AKT signaling pathway (4, 5). It is now well established that PTEN plays a role in growth and survival. Studies in recent years also established a role of PTEN in metabolic regulation (6, 7). In this review, we summarized the roles of PTEN as both a tumor suppressor and a metabolic regulator and reviewed the biological functions of PTEN and its downstream target proteins. We also summarized the regulations of PTEN transcriptionally, post-transcriptionally and through regulation of its subcellular localization.

## PTEN function and its regulation

### PTEN: a dual phosphatase

**Figure 1:**
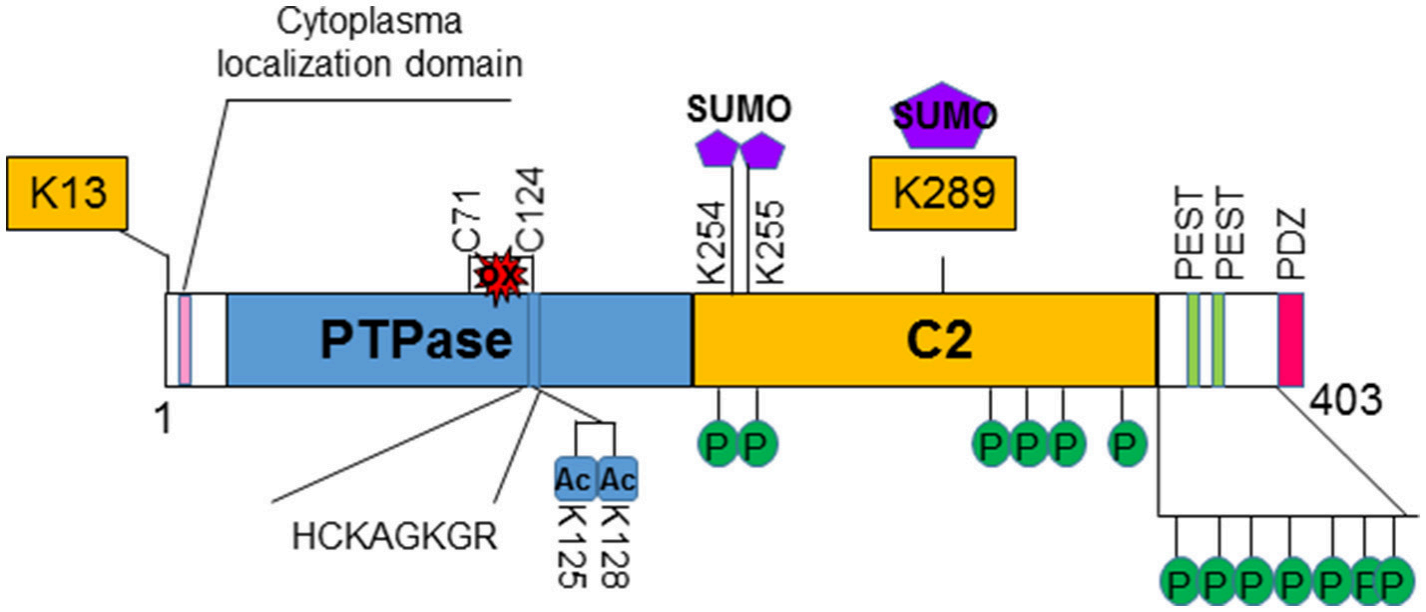
Structure and regulation of PTEN. PTEN is a 403-amino acid protein that shares sequence homology with tensin and auxilin at its amino-terminal. The hosphatase domain (PTPase) contains the signature CX5R (HCKAGKGR) p-loop structure for phosphatases. The C2 domain contains the affinity for phospho-lipids. PTEN also contains two PEST domains and a PDZ domain that may regulate its stability and subcellular localization, respectively. Several post-translational modification sites are discovered on PTEN. Two lysine sites (K13 and K289) are found to be ubiquinated and affects the localization and stability of PTEN. K289 is also sumoylated. Two additional sumoylation (K254 and K255) sites are identified that facilitates the binding of PTEN to the membrane. PTEN is also acetylated on K125 and K128 that decreases the ability of PTEN to inhibit PI3K/AKT activity. Oxidation of PTEN can occur that leads to formation of disulfide bond between C71 and C124 which results to reduced PTEN activity. Clusters of phosphorylation sites are found on PTEN. Phosphorylations of PTEN in general increases its stability but reduces its activity.

**Figure 2:**
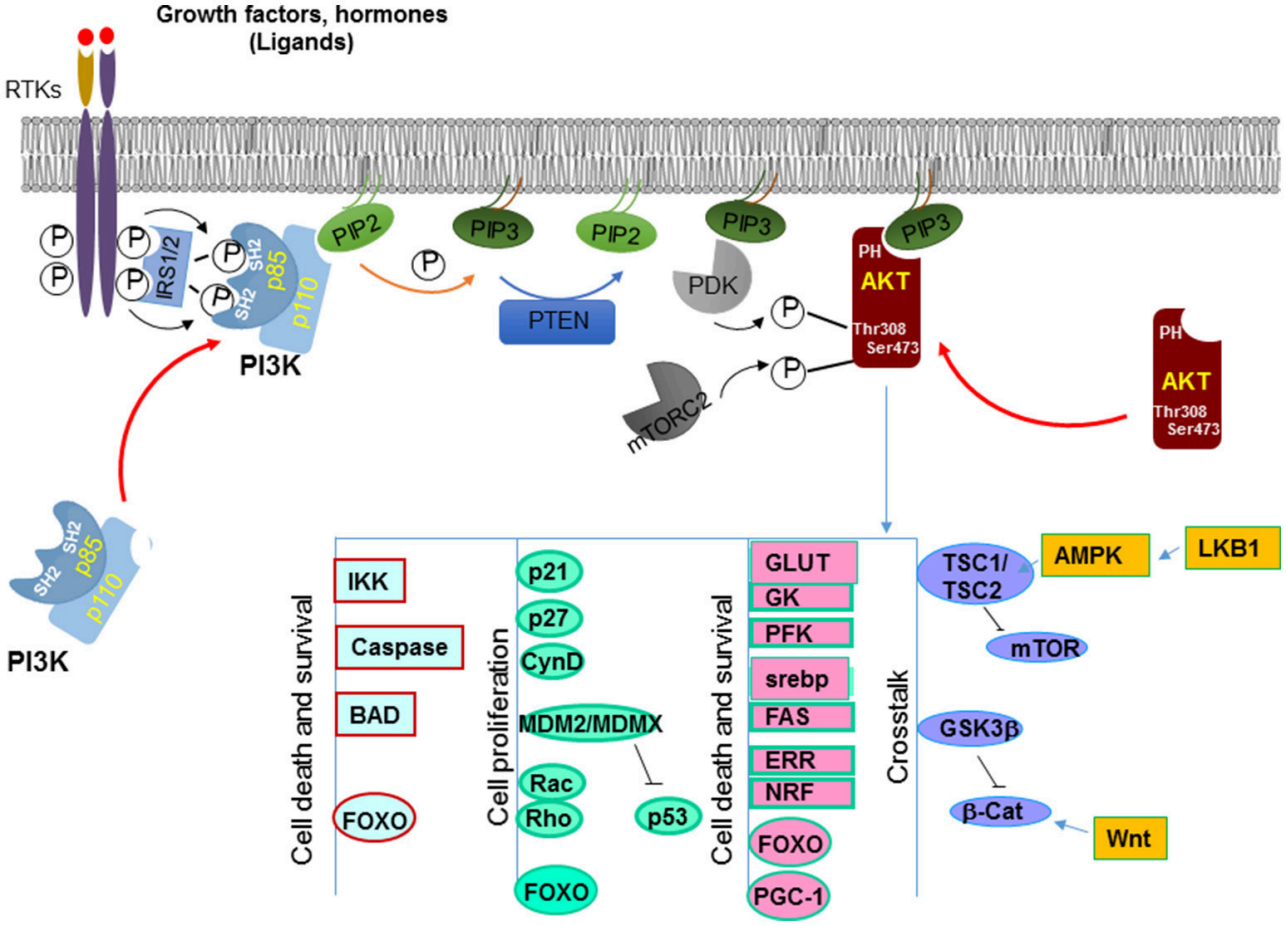
Canonical signals regulated by PTEN. PTEN dephosphorylates PIP_3_ on the 3′ position at the membrane and converts it back into PIP_2_. This action antagonize that of PI3K which adds the phosphate onto the 3′ position, leading to increased PIP_3_ production. PIP_3_ binds to the pleckstrin homology (PH) domain of downstream proteins (e.g., AKT) and provides a lipid moiety and recruits these proteins to the plasma membrane. AKT is one of the best characterized target of PI3K. Accumulation of PIP_3_ allows recruitment of AKT to the plasma membrane via direct interaction with its PH domain. AKT is then phosphorylated by two kinases, PDK1 and mTORC2 that leads to its full activation. Activated AKT has a plethora of downstream targets that it phosphorylates. Together these molecules are involved in cell growth and survival, metabolism and crosstalk with a number of other signaling pathways.

PTEN is encoded on chromosome 10q23, a region where loss of heterozygosity frequently occurs in various types of cancer (8). The protein encoded by *PTEN* contains 403-amino acid where the amino-terminal region shares sequence homology with the actin filament capping protein TENSIN and the putative tyrosine-protein phosphatase AUXILIN (1, 6). Crystal structure of PTEN revealed a C2 domain that contains the affinity for phospho-lipids on membrane and a phosphatase domain that contains the CX5R signature motif for phosphatases (9) (Figure 1). *In vitro*, PTEN is capable of dephosphorylating phospho-peptides as well as phospho-lipids. Thus, PTEN is a dual lipid and protein phosphatase. The biological effects of PTEN, however are dominated by its ability to dephosphorylate the lipid substrate phosphatidylinositol-3, 4, 5-triphosphate (PIP_3_) whereas protein substrates for PTEN are still being discovered (5, 7, 10). The lipid phosphatase motif of PTEN dephosphorylates PIP_3_ at the 3′ position and converts it back into PIP_2_ (5), leading to reduced PIP_3_ production and signals that depends on PIP_3_ (11) (Figure 2). PI3K functions to catalyze the reaction from PIP_2_ to PIP_3_. It achieves this task by phosphorylating the hydroxyl group of the 3rd position on the inositol ring of Phosphatidylinositols (12). The enzymatic function of PTEN thus acts as a negative regulatory signal for the PI3K mitogenic signaling pathway (Figure 2).

While the lipid substrate is well characterized to be PIP_3_, the identity of the protein substrates for PTEN *in vivo* has been illusive (13). However, *in vitro* study has revealed that PTEN is able to regulate cell migration by dephosphorylating itself, providing insights for investigating potential protein substrates for PTEN (14).

### Regulation of PTEN

#### Localization of PTEN

Several non-canonical nuclear localization domains have been found on PTEN (15). A cytoplasmic localization signal has been identified for the N-terminus of PTEN that spans the residue from 19 to 25 (16). Mutations in these residues leads to increased nuclear localization of PTEN with unknown mechanisms. Studies suggest that ubiquitination controls the shuttling of PTEN between cytosol and nucleus (17–19). Monoubiquitination of lysine 289 (K289) is necessary for PTEN to move into the nucleus. Mutation of this site, K289E, is found in familial Cowden's syndrome that carries multiple mutations of the *PTEN* gene (17, 20, 21). A second ubiquitination site K13 and several other sites may also facilitate the nuclear transportation of PTEN (Figure 1). In addition, the localization of PTEN is regulated by Ca2^+^-mediated interactions with the major vault protein (MVP) (22, 23). In the nucleus, PTEN is more stable and still capable of inhibiting AKT and inducing cell death. Structural analysis also reviewed that PTEN harbors a PDZ domain and two PEST sequences in the C-terminal region (24). The PDZ domain is thought to regulate PTEN's subcellular localization whereas the two PEST sequences regulate its protein stability (25) (Figure 1).

Interestingly, while monoubiquitination leads to its nuclear shuttling, poly-ubiquitination of PTEN leads to its degradation. Though disagreement exists, an E3 ubiquitin ligase for PTEN has been reported (18, 21). NEDD4-1 was reported to add ubiquitin to both K13 and K289 of PTEN molecule, leading to both mono- and poly-ubiquitination of PTEN. However, others suggest that NEDD4 is dispensable for the regulation of PTEN (21).

#### Transcriptional regulation of PTEN

PTEN is also regulated on the transcriptional and post-transcriptional levels. Several transcriptional factors have been reported to control the transcription of PTEN, including the tumor suppressor p53, the early growth response protein 1 (EGR-1), a metabolic regulatory gene peroxisome proliferation-activator receptor γ (PPARγ) [for detail, see 6)] and active transcription factor 2 (ATF2) (26). PTEN is also transcriptionally repressed by SNAIL and SLUG (27). These two zinc finger-like transcriptional factors compete with p53 for PTEN promoter binding. In addition, the nuclear factor kappa B (NFkB), the AP-1 transcription factor subunit c-Jun and the Notch signaling coregulatory CBF-1 (C-promoter binding factor-1) also bind to the *PTEN* promoter to regulate its transcription (28, 29).

More recently, regulation of PTEN by RNA-RNA interaction is reported that include microRNAs and long noncoding RNAs. Several miRNA including miR-205, miR-122, miR-21, etc. were identified to bind to the 3′untranslated region of PTEN mRNA. Elevated levels of many miRNAs are correlated with a concomitant reduction of PTEN mRNA (30–33). The long noncoding RNA that is encoded by the PTEN pseudogene transcript PTENP1 shares sequence identity with PTEN mRNA (34, 35). This transcript binds to miRNAs that target PTEN, leading to stabilization of PTEN mRNA. The antisense transcript of this pseudogene binds to the promoter of PTEN and negatively regulates the transcription of PTEN (34, 36).

#### Post-translational regulation of PTEN

Post-translationally, PTEN is modified by acetylation, oxidation and phosphorylation in addition to the ubiquitination discussed above [for detail, see 37)] (Figure 1). Phosphorylation of PTEN occurs on several clustered residues in the C-terminal domain of PTEN (38, 39). Several enzymes are responsible for these phosphorylations including casein kinase 2 (CK2), GSK3β, RhoA kinase, and P110δ subunits of PI3K (38–43). Phosphorylation of PTEN generally leads to the stabilization of the molecule but may reduce its activity (38, 39, 44). Recently, it was shown that ataxia-telangiectasia-mutated kinase (ATM) phosphorylates SUMOylated PTEN in response to γ-irradiation which leads to its nuclear exclusion (45). SUMOylation of PTEN typically occurs on K254 and K255 (46) which facilitates its binding to the membrane whereas modification on K289 is involved in its nuclear shuttling due to competition with ubiquitination modification. A couple of lysine residues at the catalytic domain of PTEN, lysine 125 and 128 are acetylated by PCAF (47). These acetylations lead to the diminished ability of PTEN to inhibit downstream events. PTEN is also regulated by the redox status of the cells. Two cysteine residues (124 and 71) form a disulfide bond in response to H_2_O_2_ treatment that leads to reduced activity of PTEN (48). Cys 124 is one of the “hot spots” that are often found mutated in human cancers.

## PTEN as a tumor suppressor

### Canonical signaling pathways regulated by PTEN

Accumulation of PIP_3_ serves as a major signal for growth factor stimulation. PIP_3_ binds to the pleckstrin homology (PH) domain of downstream proteins (e.g., AKT) and provides a lipid moiety and recruits these proteins to the plasma membrane (49). Binding of PIP_3_ to the PH domain also changes the confirmation of these proteins so they can later be activated by phosphorylation. By reducing the intracellular levels of PIP_3_, PTEN inhibits the activation of downstream proteins of the PI3K pathway, including the serine/threonine kinase AKT and the protein kinase C (PKC).

A well-known downstream effector protein of the PTEN signal is AKT (50), which plays a critical role in regulating a number of cellular activities including cell growth, survival, cell migration and differentiation, cell and organ size control, metabolism, et al. [for detailed review, see 51)]. AKT, also known as Protein Kinase B (PKB) is a serine/threonine kinase. Following PI3K activation, accumulation of PIP_3_ allows recruitment of AKT to the plasma membrane via direct interaction with its PH domain (11). This binding of AKT to PIP_3_ not only allows AKT to be translocated to the membrane but also exposes sites on AKT where it can be further modified. It has been shown that AKT is phosphorylated by another PH domain-containing kinase 3-phosphoinositide-dependent protein kinase 1 (PDK1) at Thr308 (13, 52). This phosphorylation on Thr308 is important for initial activation of AKT whereas phosphorylation of Ser473 by mTORC2 is required for maximal AKT activation (53) (Figure 2).

Activated AKT phosphorylates a plethora of downstream targets including the regulations of kinases such as glycogen synthase kinases (GSK3α and β) (54), IκB kinases (IKKα and IKKβ) (55), apoptotic factors such as BAD (56), MDM2, a ubiquitin ligase for p53 (57), GTPases like Rac and Rho (58), cell cycle inhibitors p21 and p27 (59), and transcription factors such as forkhead transcription family (FoxO) members (Figure 2) (60–62). Phosphorylation by AKT regulates the functions of these molecules that are important for multiple cellular processes. For instance, phosphorylation of the pro-apoptotic factors BAD, caspases 3 and 9 by AKT renders them inactive and thus promotes cell survival (56, 63, 64). Phosphorylation of p21 on T145 and p27 on T157 leads to their nuclear exclusion and the inability of these cell cycle inhibitors to inhibit cell proliferation (65, 66). Likewise, AKT also directly phosphorylates MDM2 and MDMX (67, 68). The phosphorylated MDM2 and MDMX bind to 14-3-3 proteins, leading to stabilization of the MDM2-MDMX complexes which mediates the degradation of p53 to keep the level of p53 low in the cells.

AKT also phosphorylates forkhead transcriptional factors and induces their binding to 14-3-3 proteins (63). This process blocks their translocation to the nucleus. Several members of the forkhead transcriptional factor family are targets of AKT, including FOXO1 and FOXO3. The binding elements for these forkhead transcriptional factors are widely spread on promoter regions of genes that regulate cell proliferation, survival and metabolic changes (69). For example, FOXO3a binds to the promoters of Bim and PUMA and can initiate apoptosis cascades by inducing the transcription of these death genes (70, 71). FOXO1 transcriptionally activates p21 and p27 and inhibits cell proliferation through these actions (72, 73). Furthermore, these forkhead transcriptional factors are also responsible for many metabolic effects induced by insulin signaling through the PI3K/AKT signaling pathway (74). Additional evidence suggests that the forkhead transcriptional factors may play a key role in the feedback regulation of the Insulin/PI3K/AKT signaling pathway (75).

Two substrates of AKT, GSK3β and tuberous sclerosis complex TSC1/2, play important roles in mediating cross talks between PI3K/AKT signaling pathway and other signaling pathways (Figure 2). GSK3β is phosphorylated by AKT on Serine 21/9 which inhibits its activity (76). GSK3β is an important regulator in Wnt signaling. It phosphorylates β-catenin, resulting in its ubiquitin-mediated degradation. The crosstalk between PTEN and Wnt signaling may underlie some of the effects of PTEN on the regulation of stem cell maintenance (77–79) and G_0_-G_1_ cell cycle regulation (80–84). Another substrate of AKT is TSC1/2. TSC1/2 plays a key role in incorporating metabolism and cell size control, together with cell growth and proliferation regulation (85). The heterodimer of TSC1 and TSC2 is essential for suppressing the function of mTOR (mammalian target of rapamycin). TSC2 activity is inhibited when phosphorylated by AKT (86). Therefore, by acting on TSC2, AKT induces the activity of mTOR and the downstream events of mTOR activation that include metabolic changes, protein translation as well as cell proliferation. This regulation of mTOR by AKT-TSC-mediated signal allows the crosstalk of PTEN with another tumor suppressor LKB1 (87).

### Nuclear PTEN

**Figure 3:**
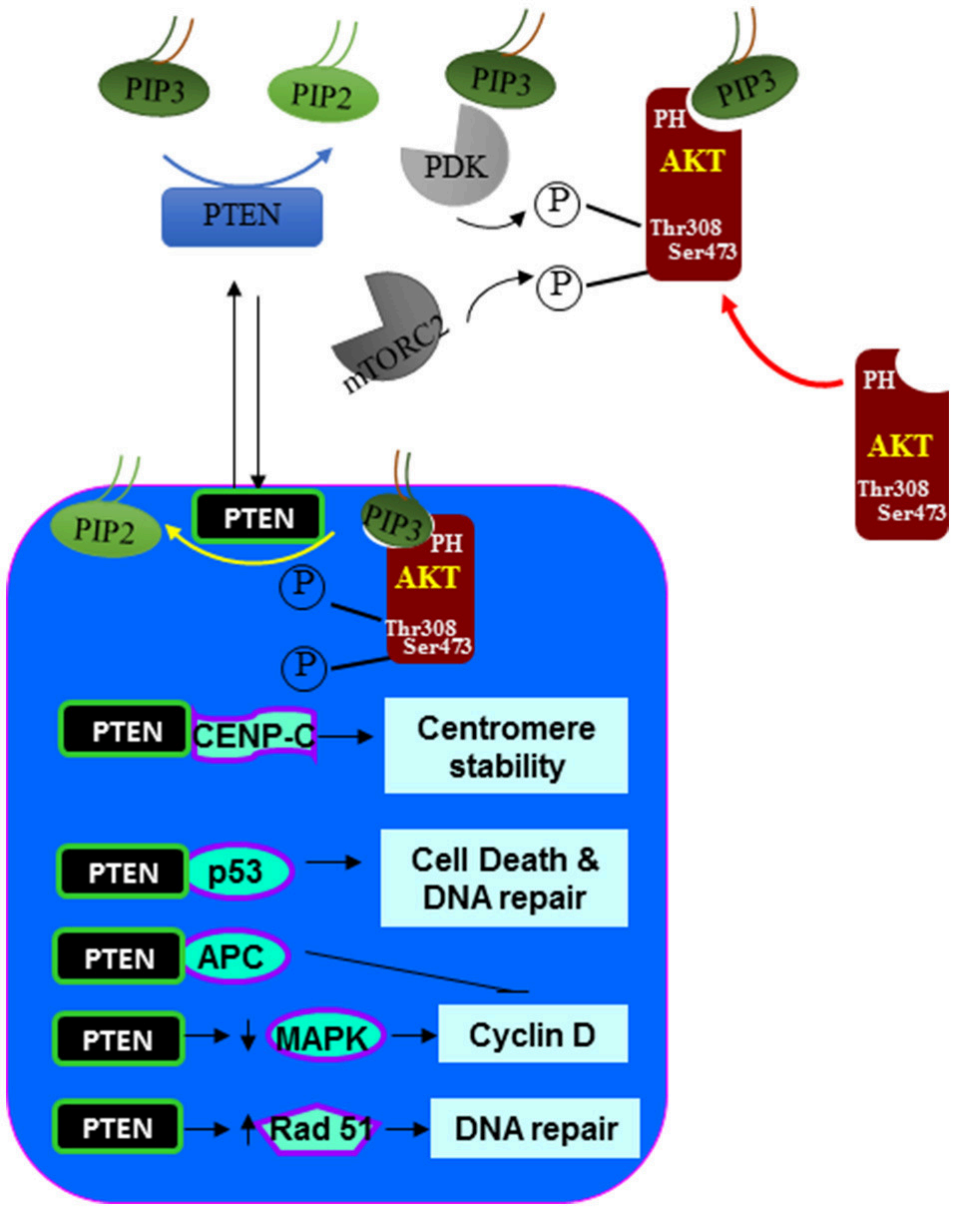
Nuclear signals regulated by PTEN. In addition to the dephosphorylating PIP3 at the plasma membrane. PTEN is also found in the nucleus. In the nucleus, PTEN can act similarly as it does at the plasma membrane by inhibiting the function of AKT. In addition, PTEN also associate with a number of nuclear proteins and regulate other cellular functions such as centromere stability, DNA repair, cell death and proliferation.

In earlier studies, PTEN was reported to be a protein that is exclusively localized in the cytoplasm. However, it is clear now that PTEN can be both cytoplasmic and nuclear (88–90). In more differentiated and resting cells, PTEN is often found in the nucleus even though it was originally identified to be a cytosolic protein (using primarily tumor cells) (90). Nuclear PTEN also plays other roles in addition to its lipid phosphatase activity and the nuclear function of PTEN is important for the ability of PTEN to inhibit tumor development (Figure 3). Nuclear PTEN is reported to play important roles in chromosome stability, DNA repair and cell cycle regulation. In the nucleus, PTEN promotes the stability and transcriptional activity of the tumor suppressor p53 by directly associating with p53 (91–93). Forced expression of PTEN in the nucleus led to MAP kinase-dependent inhibition of cyclin D1 expression (15, 22). Nuclear expression of PTEN results in the dephosphorylation of MAP kinase. Whether this is a direct effect of the protein phosphatase activity of PTEN is not clear. In addition, PTEN is found to be associated with the centromere in the nucleus by direct binding to the centromere specific binding protein C (CENP-C) (94). Disruption of this binding leads to premature centromere separation. In addition, PTEN is also found to collaborate with E2F to induce the expression of Rad51 and thus enhance DNA repair (94). This relationship between PTEN and Rad51 may explain the observation that double-stranded DNA breakage rate is found to be increased when nuclear PTEN function is interrupted. PTEN also interacts with the anaphase-promoting complex (APC) to promote its association with its binding partner which together results in proteolysis of mitotic cyclins (95).

## PTEN as a metabolic regulator

As an important growth and survival regulatory gene, germline deletion of *Pten* in mice was shown to be embryonic (96–98). Heterozygous mice develop hamartomatous polyps in the colon and tumors in multiple other tissues (99). In human, germline PTEN mutation leads to a number of familial diseases that are characterized by multiple hamartomatous lesions and predisposition to cancer development (100). Conditional deletion of *Pten* in mouse models has been done in multiple organs. Collectively, these studies confirm the signaling studies verifying PTEN as a tumor suppressor that regulates cell growth and survival. These studies have been comprehensively reviewed previously (6, 101).

**Figure 4:**
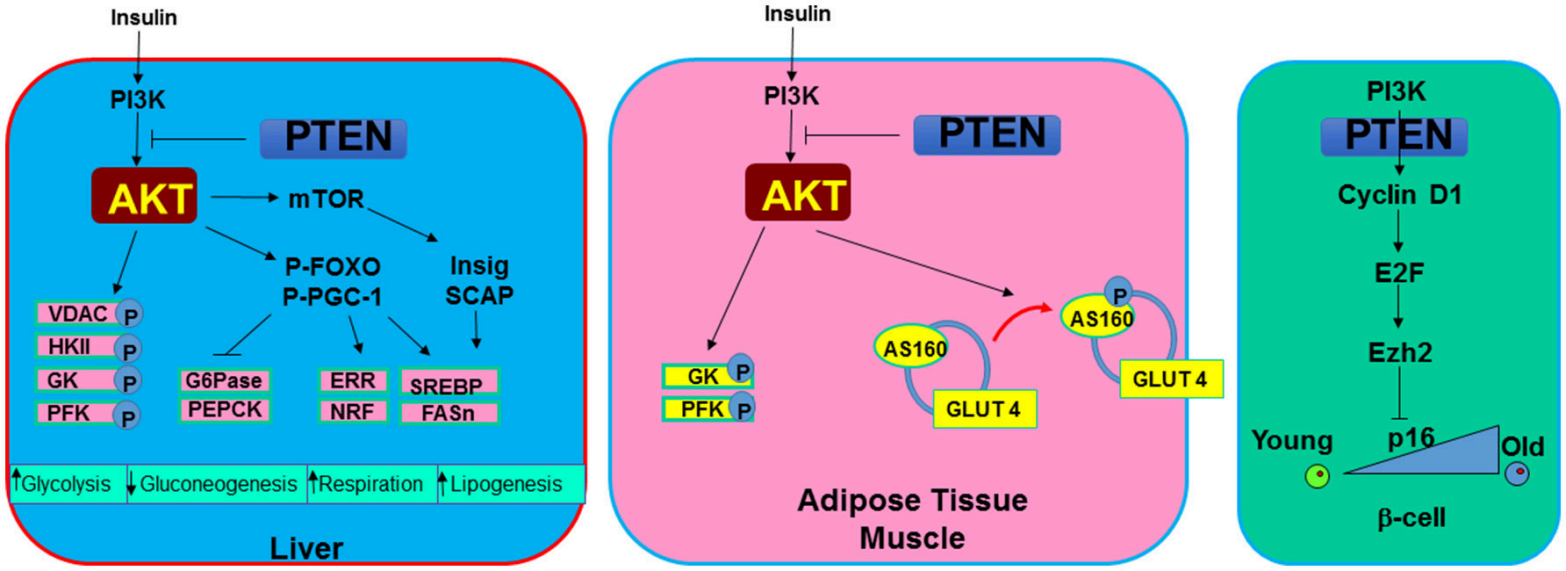
Metabolic signals regulated by PTEN. In metabolic tissues, PTEN and PI3K/AKT signal mediates the action of insulin. In the liver, induction of PI3K/AKT signal or PTEN loss leads to increased phosphorylation of several mitochondrial and glycolytic proteins that collectively increases glycolytic signaling. In addition, G6Pase and PEPCK, two rate limiting enzyme involved in gluconeogenesis are inhibited by the induction of AKT. Cellular respiration is induced with upregulation of PI3K/AKT due to their regulation on ERR and NRF, two mitochondrial biogenesis regulator. SREBP and FASn are induced through this signaling by insulin to induce lipogenesis. In adipose tissue and muscle, AKT also phosphorylates AS160 to mobilize glucose transporter GLUT4 to the membrane in response to hyperglycemia signals. In pancreatic β-cells. PTEN loss is found to rescue the age-onset loss of regenerating ability for β-cells. This phenotype appear to be regulated by the inhibition of PTEN on senescence regulator p16 via E2F and Ezh2 signaling.

While PTEN loss promotes tumorigenesis in multiple organs, genetic studies also indicate that PTEN loss leads to a number of metabolic changes that collectively improve overall insulin sensitivity (Figure 4). PTEN, being a major negative regulator of the PI3K/AKT signaling, is found to play an important role in both lipid and glucose metabolism as well as regulation of mitochondrial functions. Studies in *C. elegans* and *Drosophila* have demonstrated a highly conserved signal regulated by PTEN for both growth control and metabolism. In these organisms, the insulin/PI3K pathway negatively regulated by PTEN is used to control dauer formation, metabolism, and life span in response to nutrient availability (102–104).

### Regulation of glucose metabolism

Parallel signals for PTEN/PI3K have been reported for mammals as it was in *C. elegans* and *Drosophila*. Insulin and insulin-like growth factors (IGF) such as IGF-1 and IGF-2 binds to the insulin and IGF receptors. Binding of insulin and IGF to these receptors either directly induces the activation of PI3K or results in phosphorylation of insulin receptor substrate (IRS) as an adaptor protein to recruit and activate PI3K (10). Through this action and the downstream activation of AKT, adipocytes and myocytes sense the elevated insulin levels and initiate glucose uptake. The serine/threonine kinase AKT phosphorylates a 160-KDa substrate AS160 at Thr642 in adipocytes (105). Phosphorylation of this protein, identified as a GTPase-activating domain for Rab, is found to be responsible for membrane trafficking of GLUT4 induced by the insulin/PI3K signaling. In addition, AKT phosphorylates a variety of targets involved in the regulation of metabolism. Phosphorylation and inhibition of GSK3 not only contribute to regulation of β-catenin and the cell cycle, it also activates glycogen synthase (6). When PTEN is lost and GSK3 is phosphorylated, glycogen was found to accumulate in hepatocytes of the liver-specific *Pten* null mice (88). In hepatocytes, phosphorylation of FOXO by AKT blocks the transcription of glucose-6-phosphatase (G6Pase) and phosphoenolpyruvate carboxykinase (PEPCK) (74), two rate-limiting enzymes in the process of gluconeogenesis. In addition, AKT was also reported to directly phosphorylate proxisome proliferator-activated receptor gamma co-activator (PGC-1a) at S570 (106). This phosphorylation event was also found to mediate the transcriptional repression of G6Pase and PEPCK. These signals regulated by AKT and blocked by PTEN are important for how metabolic organs like the liver, muscle and adipose tissue respond to elevated insulin signals.

Consistent with these metabolic signals regulated by PTEN, deletion of *Pten* in the liver led to robust downregulation of PEPCK (88). Moderate downregulation of G6Pase was also reported. In the adipose tissue, deletion of *Pten* resulted in increased insulin sensitivity and resistance to streptozotocin-induced diabetes (107). Increased GLUT4 membrane localization on adipocytes was observed in these mice.

### Regulation of lipid metabolism

In addition to glucose metabolism, the PTEN-regulated PI3K signaling also controls lipid metabolism. The sterol receptor element binding protein, SREBP, serves as a key transcriptional factor for genes involved in the biosynthesis of fatty acids and their further incorporation into triglycerides and cholesterol. As a master transcriptional factor controlling the de novo lipogenesis process, SREBP binds to the promoters of many lipogenic enzyme genes, including fatty acid synthase (Fasn) and acetyl-CoA carboxylase (ACC), as well as those controlling the production of NADPH, a reducing equivalent needed for lipid biosynthesis. The PTEN/PI3K/AKT signaling-controlled SREBP expression is mediated through multiple levels including transcriptional and post-translational processing of SREBP. The downstream target of AKT, the forkhead transcriptional factor, FoxO1 regulates SREBP and lipogenesis by repressing SREBP transcription (108). Interestingly, the function of FOXO1 on Fasn expression is dependent on whether PI3K/AKT signal is induced (109). Using rapamycin and siRNA to inhibit mTORC1 and other signals involved in the AKT pathway, it was shown that transcription induction of SREBP1 and lipogenesis is also dependent on TORC1 activity (110). However, this effect was not supported by observations in the TSC1-deficient mice, defective in mTORC1 signaling, which are resistant, rather than sensitive, to high fat diet (HFD)-induced steatosis (111). The processing of SREBP is dependent on two proteins, SREBP cleavage-activating protein (SCAP) and insulin induced gene (Insig). In response to sterol demand, SCAP cleaves SREBP to produce the mature active form of transcriptional factor that moves to the nucleus. Binding of Insig to SCAP prevents this action and thus inhibits the processing of SREBP. While oxysterols suppresses the expression of Insig-1, inhibition of PI3K/AKT activity blocks this inhibition and allows the processing of SREBP (112), consistent with a role of AKT in SREBP processing. This processing is both mTORC1-dependent and mTORC1-independent (111). Thus, both PTEN/PI3K/AKT downstream signaling targets, TORC1 and FoxO1, play critical roles in controlling SREBP expression and lipogenesis. In addition, Maf-1, a central repressor of genes transcribed by RNA pol III is recently found to be regulated by PTEN through AKT2 and mTOR (113). While SREBP binds to the promoter of Fasn and positively regulates its expression, Maf-1 was shown to occupy the promoter and repress the expression of Fasn.

Consistent with these signaling analysis, loss of *Pten* in the liver led to elevated de novo lipogenesis through robust induction of SREBP and Fasn expression (88). The accumulation of lipid and elevated lipogenesis is a result of activation of AKT2 as deletion of *Akt2* completely reversed the phenotype (109, 114). This effect of AKT is both mTOR dependent and independent (111). In addition, FOXO1 gain of function has also been shown to induce lipid synthesis (115).

### Regulation of mitochondrial metabolism

In recent years, studies attempt to elucidate the molecular signals underlying “Warburg effects” have led to the discoveries of novel roles for PI3K/AKT signaling in mitochondrial function (10). In addition to regulating pro- and anti-apoptotic factors (10), AKT promotes binding of hexokinase II to the mitochondrial voltage dependent anion channel (VDAC) (116). This event, occurring at the mitochondrial outer membrane allows rapid phosphorylation of available glucose molecules and efficient conversion to ATP from glycolysis. AKT was also found to be localized in the inner membranes of the mitochondria (117, 118). In the mitochondria matrix, AKT phosphorylates mitochondrial pool of GSK3β and regulates mitochondrial respiration through phosphorylation of pyruvate dehydrogenase (PDH) (118). In addition, the mitochondrial localized AKT also plays a role in the transcription regulation of mitochondrial DNA. A FOXO3-response element has been found on the promoter of a mitochondrial encoded gene, 3-hydroxy-3-methylglutaryl-CoA (HMG-CoA) (119).

In the nucleus, the PI3K/AKT signaling controls mitochondrial gene transcription network through multiple different mechanisms. The forkhead transcriptional factor FOXO3 has been demonstrated to be a transcriptional regulator of mitochondrial genes. In colon cancer cells induced to express a constitutively active form of FOXO3a, a large number of mitochondrial genes are downregulated (120). These genes include Tfam and TFB1M&2M, the nucleus-encoded auxiliary factors for mitochondrial gene transcription. One of the global regulators of metabolism including lipid and glucose metabolism as well as mitochondrial metabolism is PGC-1 (121). As a transcriptional coactivator, members of PGC-1 family of coactivators have the ability to interact with a number of different transcriptional factors including PPARs for fatty acid oxidation, FOXO1 for lipogenesis, FOXO1 and glucocorticoid receptor (GR) for gluconeogenesis, and estrogen-related receptors (ERRs) for mitochondrial function.

The best characterized isoform of ERRs, ERRα is abundantly expressed in high oxidative organs and recognized as a key regulators of adaptive energy metabolism (122). ERRα, itself, is a weak transcriptional factor. Both the activity and expression of ERRα are significantly increased when physically bound by PGC-1α (123). AKT activation was found to control mitochondrial gene transcription by phosphorylating and activating CREB transcriptional factor independent of the cAMP mediated activation of PKA, the common signal that induces CREB phosphorylation (124). When phosphorylated, CREB induces the transcription of PGC-1. Thus, in addition to removing the inhibition of FoxO and phosphorylating PGC-1, activation of AKT also positively induces PGC-1 transcription by phosphorylating CREB. Being the coactivator, PGC-1 robustly increases the transcriptional activity of ERRα to promote transcription of genes encoding mitochondrial function, including TFAM, TFB1M&2M and medium-chain acyl-coA dehydrogenase (MCAD) (124). In hepatocytes, this induction of ERRα leads to increased oxygen consumption and elevated ROS production, likely contributed to the liver injury (and lipid accumulation) phenotypes observed with *Pten* loss in the liver.

In addition, the AKT substrate consensus sequence has been found on NRF1, another gene involved in mitochondrial gene transcription. In H4IIE hepatoma cells, phosphorylation of NRF1 by AKT is reported to mediate pro-oxidant t-BOOH induced Tfam expression (125). Thus, through directly phosphorylating FOXO and NRF1 or indirectly inducing ERRα expression, AKT controls the gene transcriptional networks of mitochondria. Consistently, over-expression of NRF1 and AKT has been shown to mimic the effect of TFAM to abrogate 1-methyl-4-phenyl-2, 3-dihydropyridinium ion induced mitochondrial damage (126), confirming a signaling relationship between PI3K/AKT/FOXO signal and mitochondrial gene transcription regulation.

## Paradoxical roles of PTEN regulated metabolic and growth signals on tumor growth and metabolism

### Metabolic sensitivity regulated by PTEN

In mammals, ectopic expression of PTEN by introduction of bacterial artificial chromosomes (BACs) into the mouse genome led to reduced body size, increased energy expenditure and low body fat content (127, 128). Consistent with the observations in *C. elegance* and *Drosophila*, these mice also have a longer tumor free lifespan. This enhanced metabolic phenotype however is paradoxical with the enhanced metabolic functions associated with PTEN loss observed with the tissue specific *Pten* deletion mice. In adipose tissue, liver, pancreatic β-cells and muscle, deletion of *Pten* consistently lead to enhanced insulin and metabolic sensitivity as well as resistance to HFD induced diabetes (88, 89, 107, 129–131). In addition to the enhanced ability to transport and metabolize glucose by adipocytes, myocytes and hepatocytes, PTEN loss was associated with enhanced energy expenditure in brown adipose tissue (128). In β-cells, deletion of *Pten* relieved the suppression of cell cycle re-entry by inhibiting the senescence regulatory gene p16^Ink4a^ through E2F/Ezh2 mechanism (132). This regulation led to the rescue of aging-induced loss of growth potential in β-cells. The enhanced ability of pancreatic islets to respond to hyperglycemic stress led to improved systemic metabolic health (89, 130). Overall, PTEN loss and activation of PI3K/AKT signal lead to the improved ability to handle metabolic stress in mice. The improved metabolic health phenotypes observed with overexpression of PTEN is likely contributed to metabolic adaptation.

### Tumor metabolism regulated by PTEN

While loss of PTEN leads to improved insulin sensitivity, this metabolic effect has been credited for the tumor suppressing functions of PTEN (7). The metabolic signals regulated by this pathway, including the glycolytic signal such as localization of glucose transporters, activation of hexokinase and phosphofructokinase as well as induction of de novo lipogenesis are among the signals that are recognized as promoting factors for tumorigenesis. Indeed, a number of metabolic enzymes, particularly glycolytic genes have been found to have oncogenic or tumor suppressive functions as manipulation of these genes modulate tumor growth. Particularly, expression of isoform specific metabolic genes appears to be linked to tumorigenesis (133). Association of lipogenic and other lipid metabolic genes with tumors are recognized but more works are needed to understand their contributions to tumorigenesis.

### Steatosis due to PTEN loss establishes a tumor promoting environment

During insulin resistance, suppression of hepatic glucose synthesis by insulin is blunted and the persistence of hepatic glucose output leads to postprandial hyperglycemia (134). At the same time, hyperinsulinemia signal in the liver induces lipogenesis, resulting in fatty liver disease that is a hallmark of insulin resistance syndrome. This differential response of gluconeogenesis and lipogenesis to insulin during insulin resistance has been termed “selective hepatic insulin resistance” (135). Mimicking insulin signal in the liver, loss of hepatic PTEN resulted in non-alcoholic steatohepatitis (NASH) while suppressing gluconeogenesis (89, 109, 136, 137). Unlike that observed with insulin resistance, NASH developed in the liver-specific *Pten* deletion mice is not due to hyperinsulinemia resulting from high circulating glucose levels. Locally enhanced hepatic PI3K/AKT signal actually led to improved ability for the liver to handle glucose, turning the liver into a glucose sink, leading to an improved ability to handle glycemic stress in these mice.

**Figure 5:**
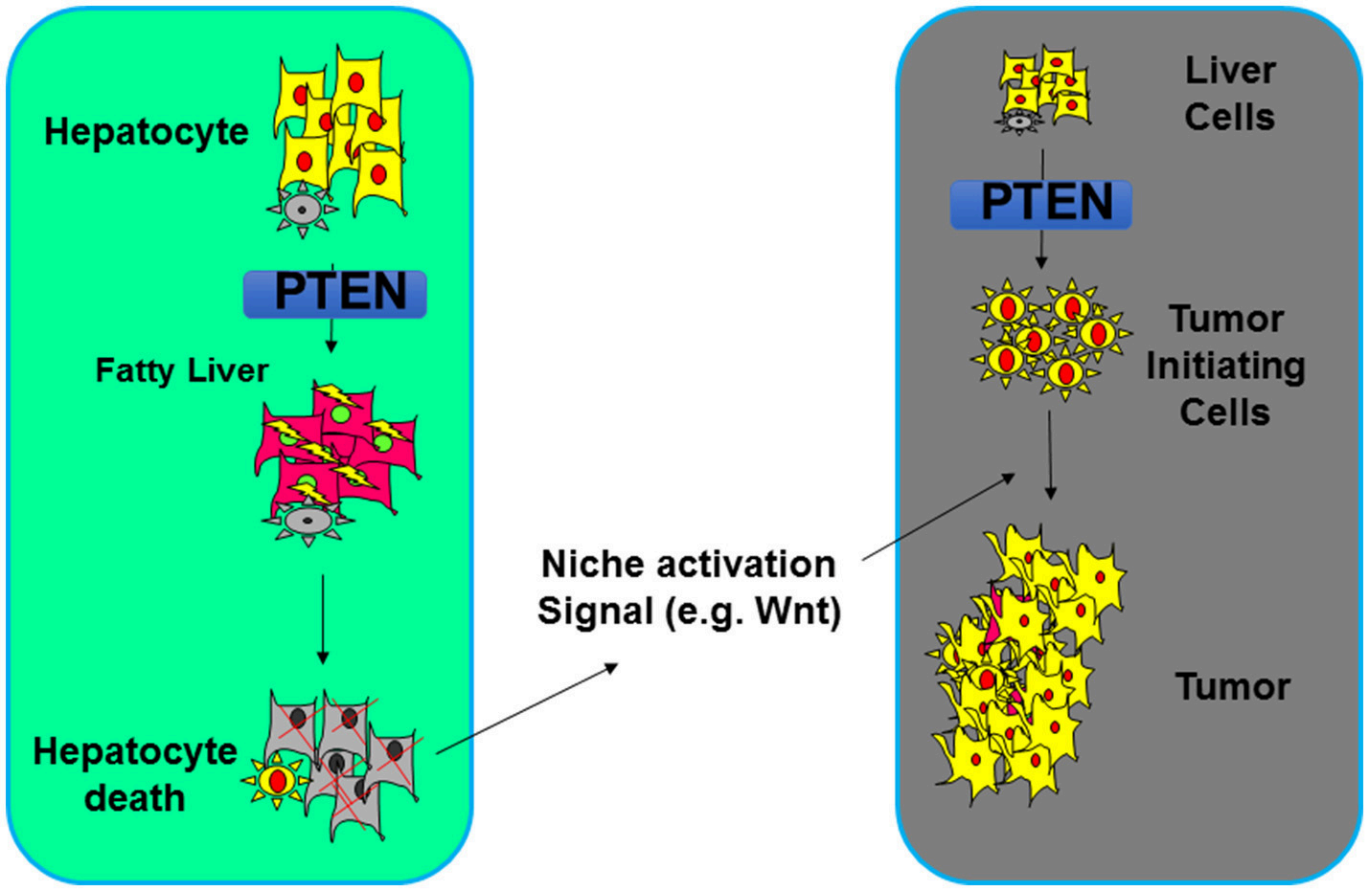
Steatosis regulation by PTEN alters the tumor microenvironment. Deletion of *Pten* leads to increased lipogenesis and deposition of lipid. In hepatocytes, such lipid accumulation results in hepatocytes death which establishes a niche activation signal (e.g., Wnt). This then leads to the proliferation of tumor initiating cells and tumorigenesis.

While the increased insulin/PI3K/AKT signal in the liver leads to improved systemic insulin sensitivity (88), the resulting NASH due to increased de novo lipogenesis however forms an environment that results in damage of the liver parenchymal (138–142). When NASH is inhibited via either dietary approach or genetic deletion of a metabolic AKT, *Akt2*, tumor development is inhibited (79, 114). The NASH thus serves as a tumor promoting event that promotes the development of tumors that arose from the PTEN loss transformed tumor-initiating cells (77–79, 114). How steatosis establishes a tumor environment is being explored currently. In the liver, inflammation as a result of damage to the liver parenchymal was shown to play an important role (Figure 5). Wnt signal produced by macrophages is one of the niche signals established by this NASH environment to promote tumorigenesis (79).

## Future considerations

PTEN is a critical regulator of cell growth/survival as well as metabolism. As a metabolic regulator, PTEN controls the metabolism of both glucose and fatty acids. These effects of PTEN through targeting the PI3K/AKT dependent and independent pathways lead to suppressed insulin sensitivity and inhibited cell growth and survival. While the signals by which PTEN regulates growth and survival has been well elucidated, the mechanisms by which PTEN regulates metabolism, particularly lipid and mitochondrial metabolism is not well understood. Future studies to understand the molecular signals that PTEN controls to regulate these cellular functions are necessary for both the cancer and diabetes treatment.

## Author contributions

C-YC and JC drafted the original manuscript. LH proofed the manuscript. BS edited the finalized the manuscript.

### Conflict of interest statement

The authors declare that the research was conducted in the absence of any commercial or financial relationships that could be construed as a potential conflict of interest.
